# Risk of second breast cancers after lobular carcinoma in situ according to hormone receptor status

**DOI:** 10.1371/journal.pone.0176417

**Published:** 2017-05-03

**Authors:** Kai Mao, Yaping Yang, Wei Wu, Shi Liang, Heran Deng, Jieqiong Liu

**Affiliations:** 1 Guangdong Provincial Key Laboratory of Malignant Tumor Epigenetics and Gene Regulation, Department of General Surgery, Sun Yat-sen Memorial Hospital, Sun Yat-sen University, Guangzhou, China; 2 Guangdong Provincial Key Laboratory of Malignant Tumor Epigenetics and Gene Regulation, Department of Breast Surgery, Breast Tumor Center, Sun Yat-sen Memorial Hospital, Sun Yat-sen University, Guangzhou, China; University of North Carolina at Chapel Hill School of Medicine, UNITED STATES

## Abstract

**Background:**

Although subsequent breast cancer risk after primary lobular carcinoma in situ (LCIS) has been studied intensively, whether the risk of second breast cancer after first LCIS varies with hormone receptor (HR) status of primary tumor remains unclear.

**Methods:**

We identified 10,304 women with primary pure unilateral LCIS between 1998 and 2007 from the Surveillance, Epidemiology and End Results (SEER) 18 Registries. Kaplan–Meier estimates of 5 or 10-year probabilities of second ipsilateral breast cancers (IBCs) and contralateral breast cancers (CBCs) were calculated. Multivariable Cox proportional model was performed to identify impact of HR status of primary LCIS, and other demographic, clinicopathologic or treatment characteristics on risk of second IBCs or CBCs.

**Results:**

Of the 10,304 women with primary LCIS included in this study, 9949 (96.5%) patients had HR+ tumors, and 355 (3.5%) had HR- tumors. Multivariable-adjusted analyses showed that although there was no difference in risk of total second IBCs between women with HR+ and HR- LCIS (*P* = 0.152), patients with HR+ LCIS had a statistically lower risk of second invasive IBCs compared to those with HR- LCIS (hazard ratio 0.356, 95% CI 0.141–0.899, *P* = 0.029). Women with primary HR+ LCIS had lower risks of both second total and invasive CBCs compared to those with HR- LCIS (total CBCs: hazard ratio 0.340, 95% CI 0.228–0.509, *P*<0.001; invasive CBCs: hazard ratio 0.172, 95% CI 0.108–0.274, *P*<0.001). Additionally, black women had a 2-fold risk of developing subsequent total IBCs than white women (*P* = 0.028).

**Conclusions:**

This population-based study demonstrated that the risk of second breast cancers was significantly increased in women with HR- first LCIS compared to those with HR+ LCIS. These findings warrant intensive surveillance for second breast cancers in HR- LCIS survivors.

## Introduction

First reported in 1941[1], an increase in lobular carcinoma in situ (LCIS) incidence has been described, from 2.0 per 100,000 women in the year 2000 to 2.75 per 100,000 in 2009 across the United Sates [2]. Unlike ductal carcinoma in situ (DCIS), LCIS is typically confined to lobules and terminal ducts of the breast and is usually found incidentally in biopsy specimens[3]. Women with LCIS showed a 7 to 10 fold increase in the risk of developing subsequent breast cancers compared with the general population [4–6], and LCIS women have significantly higher incidence rates of second invasive breast cancer andcontralateral breast cancer than women with DCIS[7,8]. Modern management of LCIS includes surveillance, risk reduction via chemoprevention, and bilateral prophylactic mastectomy.

Breast cancer is recognized as a heterogeneous group of malignancies, and hormone receptor (HR) status of tumor is correlated with substantial variation in breast cancer incidence, as well as survival rates[9]. Similar to invasive lobular breast cancer, LCIS also has different subtypes according to biomarker profiling such as HR status[10]. Approximately 96% to 98% LCIS have been reported to be HR positive [11–13]. Prior studies have demonstrated that the risk of second contralateral breast cancer after first primary invasive breast cancer varied with HR status of primary tumor; they found that HR negative invasive ductal or lobular breast cancer women had a significantly higher risk of developing second contralateral breast cancer than women with HR positive invasive breast cancer [14–16]. Although subsequent breast cancer risk after primary LCIS has been studied intensively [8,6,7,5,4,17], whether the risk of second breast cancer after first primary LCIS varies with HR status of primary tumor remains unclear so far. Therefore, here we quantified risks of second breast cancers among LCIS survivors according to HR status in a large cohort of women with LCIS diagnosed between 1998 and 2007 in the National Cancer Institute’s Surveillance, Epidemiology and End Results (SEER) 18 registries, controlling for age at diagnosis, calendar year of diagnosis, clinicopathogical characteristics, and treatment patterns.

## Materials and methods

### Study participants

We analyzed primary pure unilateral LCIS (International Classification of Diseases for Oncology, 3rd edition [ICD-O-3] histology codes 8520)female patients diagnosed between January 1, 1998 and December 31, 2007 with no cancer history that were reported in the SEER 18 Registries. The National Cancer Institute’s SEER program collects information on cancer incidence, survival, as well as patient demographics from several geographically defined regions in the United States. We selected women between the ages of 20 and 84 who were diagnosed with LCIS and survived at least 6 months. Patients older than 84 years of age were excluded to avoid confounding influence of under-reported second breast cancers, competing medical comorbidities, and limited life expectancies. We excluded cases derived only from death certificates or autopsy. Second breast cancers diagnosed within 6 months of primary LCIS diagnosis were excluded as these were likely to be pre-existing or synchronous cancers. Patients with bilateral mastectomy were excluded as well. In addition, since women treated with unilateral mastectomy experience extremely low risk of ipsilateral breast tumors (IBTR), we excluded women with unilateral mastectomy for their first LCIS in the analysis of second ipsilateral breast cancers (IBCs)[7]. The reason we selected patients diagnosed from the year 1998 was that data on whether or not women were treated with a unilateral mastectomy or a bilateral mastectomy was available from SEER only from 1998. HR status of breast cancers was defined as follows: positive (estrogen receptor (ER) or progesterone receptor (PR) positive), negative (ER and PR negative), and unknown (ER negative and PR unknown, ER unknown and PR negative, or both ER and PR unknown); ER or PR positive groups included those with borderline results[18]. Thus, a total of 1,116 primary LCIS women with unknown HR status were further excluded. Follow-up continued until date of diagnosis of any second breast cancer, death from any cause, date of last known vital status, or end of study (December 31, 2013).

All procedures performed in studies involving human participants were in accordance with the ethical standards of the institutional and/or national research committee and with the 1964 Helsinki declaration and its later amendments or comparable ethical standards. Data within the SEER were rendered anonymous, so the study was exempt from review by the Sun Yat-sen Memorial Hospital Institutional Review Board, and no consent was needed in this study.

### Statistical analysis

Second breast cancer was defined as invasive breast cancer or breast carcinoma in situ diagnosed at least 6 months after first primary LCIS. The outcomes included second ipsilateral breast cancers (IBCs), and second contralateral breast cancers (CBCs). The demographic,clinicopathologic, and treatment characteristics were compared between patients with HR positive primary LCIS and those with HR negative tumor using chi-square test. In the SEER, some variables (eg. histologic grade) contain missing data. We considered the missing data as "unknown" for all the statistical tests. Kaplan–Meier estimates of 5 or 10-year probabilities of IBCs and CBCs were calculated, with *P* values given by log-rank test. Multivariable Cox proportional model was performed to identify the impact of HR status of primary LCIS, and other demographic, clinicopathologic or treatment characteristics on risk of second IBCs or CBCs. We used the Schoenfeld’s global test to test proportional hazards assumption of Cox model. If there were covariates not fitting the proportional hazards assumption, stratified Cox regression model will be used. Akaike information criterion (AIC) were calculated, and likelihood ratio test was used to select the best regression model. Statistical analyses were conducted using Stata 12.0 software (StataCrop, College Station, TX). All statistical tests were two-sided, and statistical significance was defined as *P*< 0.05.

## Results

### Patient characteristics

Of the 10,304 women with primary LCIS included in this study, 9949 (96.5%) patients had HR+ tumors, and 355 (3.5%) had HR- tumors. Most women (78.5%) were diagnosed after the year of 2000. Table 1 shows the demographic, clinicopathologic characteristics and treatment features for women with HR+ or HR- tumors. No difference was found between the two groups with respect to age at LCIS diagnosis, race, and laterality. The differences for year of diagnosis, histologic grade of first LCIS, first tumor size, surgery type and receiving of radiation for first LCIS between two groups of patients were statistically significant (*P*<0.05 for all comparisons), which might be partially explained by large sample size of the study. When compared with patients who had HR+ LCIS, women with HR- LCIS were diagnosed less after 2004, had higher grade (poorly or undifferentiated) tumor, larger tumor size (>2cm), received more mastectomy, and less radiotherapy.

**Table 1:** Characteristics of women with primary unilateral LCIS stratified by HR status of LCIS.

Factors	HR+ LCIS		HR- LCIS		*P* value
	No.	%	No.	%	
Age at diagnosis					0.099
Median(range)	62 (20–84)		63 (20–84)		
<50	1522	15.3	45	12.7	
50–69	5122	51.5	174	49.0	
≥70	3305	33.2	136	38.3	
Race					0.430
White	8872	89.2	307	86.5	
Black	564	5.7	24	6.8	
Other	486	4.9	23	6.5	
Unknown	27	0.3	1	0.3	
Year of diagnosis					0.009
1998–2000	2127	21.4	94	26.5	
2001–2004	4187	42.1	157	44.2	
2005–2007	3635	36.5	104	29.3	
Laterality					0.341
Left	5097	51.2	191	53.8	
Right	4852	48.8	164	46.2	
Histologic grade					<0.001
Well	2330	23.4	50	14.1	
Moderately	4157	41.8	131	36.9	
Poorly/undifferentiated	857	8.6	67	18.9	
Unknown	2605	26.2	107	30.1	
Tumor size					0.021
≤2cm	6755	67.9	219	61.7	
2-5cm	2695	27.1	120	33.8	
>5cm	439	4.4	16	4.5	
Unknown	60	0.6	0	0	
Surgery for first LCIS					<0.001
No surgery	34	0.3	5	1.4	
BCS	6354	63.9	196	55.2	
Mastectomy	3561	35.8	154	43.4	
Radiation for first LCIS					<0.001
No	4095	41.2	181	51.0	
Yes	5654	56.8	164	46.2	
Unknown	200	2.0	10	2.8	

Abbreviations: HR, hormone receptor; LCIS, lobular carcinoma in situ; BCS, breast-conserving surgery

### IBCs

**Fig 1:**
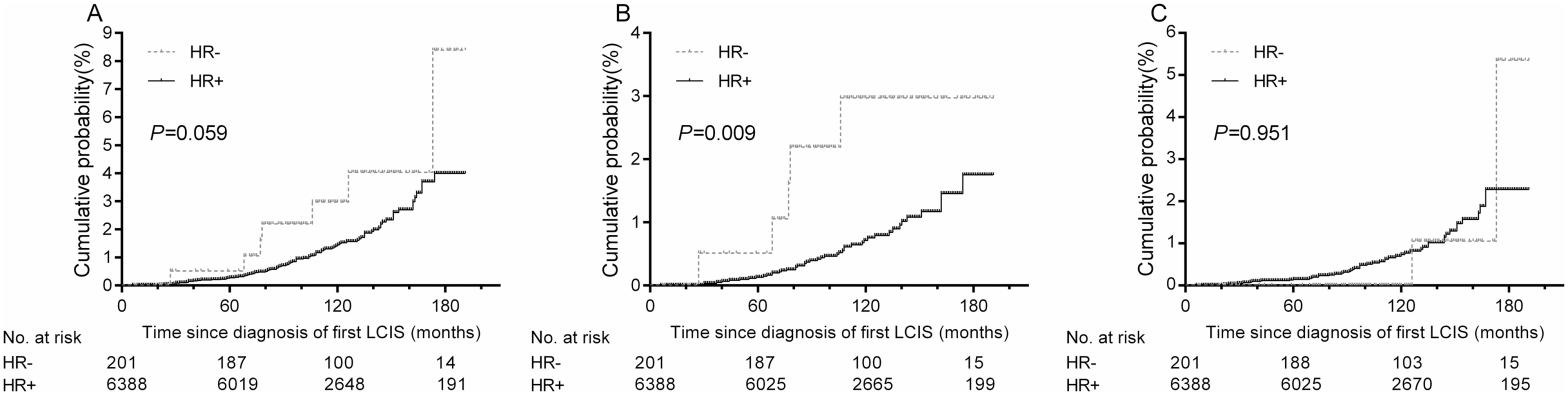
Cumulative incidences of A) total second breast cancers, B) second invasive breast cancers, and C) second breast carcinomas in situ in the ipsilateral breast in women with HR+ and HR- primary LCIS.

In the analysis of second IBCs, we excluded women with unilateral mastectomy for their first LCIS because women treated with unilateral mastectomy experience extremely low risk of IBTR. Among 6589 women treated with breast-conserving surgery or with no surgical therapy, 100 (1.5%) developed IBCs during a median follow-up of 109 months (range 6–191 months). Among these IBCs, 49 (49.0%) were invasive cancer, and 51 (51.0%) were carcinoma in situ. There was a difference in the cumulative incidence of IBCs between women with HR+ and HR- primary LCIS, although it is only marginal statistically significant: the 5-year and 10-year rates were 0.3% and 1.4%, respectively in women with HR+ LCIS, compared with 0.5% and 3.0%, respectively in those with HR- LCIS (Fig 1A, *P* = 0.059). We further analyzed whether HR status of primary LCIS was differentially associated with types of IBCs. We found that patients with HR- LCIS had a significantly higher risk of second invasive IBCs compared to those with HR+ LCIS (Fig 1B, *P* = 0.009), while there was no statistical difference in risk of second ipsilateral carcinoma in situ between women with HR+ and HR- primary LCIS (Fig 1C, *P* = 0.951).

Multivariable-adjusted analyses showed that although there was no difference in risk of total second IBCs between women with HR+ and HR- primary LCIS (Table 2, *P* = 0.152), patients with HR+LCIS had a statistically lower risk of second invasive IBCs compared to those with HR- LCIS (Table 2, hazard ratio 0.356, 95%CI 0.141–0.899, *P* = 0.029). Not surprisingly, receiving of surgical treatment and radiotherapy significantly correlated with a lower risk of total or invasive IBCs (Table 2), and young age (< 50 years) was associated with a higher risk of second total or invasive IBCs (Table 2). Black women had a 2-fold risk of developing subsequent total IBCs than white women (Table 2, *P* = 0.028). Interestingly, women diagnosed with LCIS after the year 2000 showed significantly lower risks of total IBCs and invasive IBCs compared with those who was diagnosed between 1998 and 2000 (Table 2).

**Table 2:** Impact of HR status of first primary LCIS, and other demographic, clinicopathologic or treatment characteristics on risks of second ipsilateral breast cancers by multivariable-adjusted analyses^#^ (n = 6589).

Factors	Total IBC			Invasive IBC		
	Hazard ratio	95% CI	*P*	Hazard ratio	95% CI	*P*
HR						
Negative	Reference			Reference		
Positive	0.569	0.263–1.231	0.152	0.356	0.141–0.899	0.029
Age at diagnosis						
<50	Reference			Reference		
50–69	0.453	0.282–0.729	0.001	0.422	0.217–0.821	0.011
≥70	0.609	0.360–1.029	0.064	0.514	0.244–1.082	0.080
Race						
White	Reference			/	/	/
Black	2.062	1.083–3.925	0.028	/	/	/
Other	0.636	0.200–2.019	0.443	/	/	/
Unknown	<0.001	/	1.000	/	/	/
Year of diagnosis						
1998–2000	Reference			Reference		
2001–2004	0.645	0.409–1.017	0.059	0.496	0.267–0.924	0.027
2005–2007	0.415	0.202–0.855	0.017	0.218	0.072–0.660	0.007
Histologic grade						
Well	Reference			/	/	/
Moderately	1.000	0.575–1.739	1.000	/	/	/
Poorly/undifferentiated	2.113	1.129–3.952	0.019	/	/	/
Unknown	0.977	0.544–1.756	0.938	/	/	/
Surgery for first LCIS						
No surgery	Reference			Reference		
BCS	0.079	0.036–0.172	<0.001	0.074	0.026–0.210	<0.001
Radiation for first LCIS						
No	Reference			Reference		
Yes	0.377	0.248–0.573	<0.001	0.490	0.263–0.912	0.024
Unknown	0.881	0.269–2.884	0.834	0.706	0.093–5.387	0.737

Abbreviations: HR, hormone receptor; LCIS, lobular carcinoma in situ; IBC, ipsilateral breast cancer; CI, confidence interval; BCS, breast-conserving surgery

^#^ Women who has been treated with mastectomy for their first LCIS were excluded in the analyses of second ipsilateral breast cancers.

### CBCs

**Fig 2:**
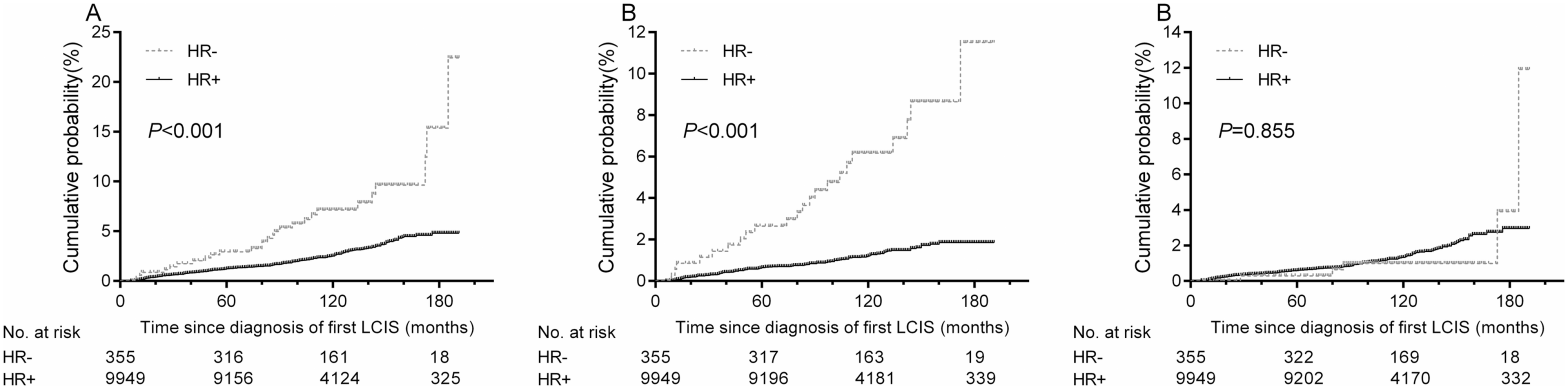
Cumulative incidences of A) total second breast cancers, B) second invasive breast cancers, and C) second breast carcinomas in situ in the contralateral breast in women with HR+ and HR- primary LCIS.

A total of 280 (2.7%) patients suffered from second CBCs among 10,304 women with primary LCIS during a median follow-up of 109 months (range 6–191 months). Of these CBCs, 137 (48.9%) were invasive cancer, and 143 (51.1%) were carcinoma in situ. There was a significant difference in the cumulative incidence of total CBCs between women with HR+ and HR- primary LCIS: the 5-year and 10-year rates were 1.2% and 2.5%, respectively in women with HR+ LCIS, compared with 3.0% and 7.2%, respectively in those with HR- LCIS (Fig 2A, *P*<0.001). We also analyzed whether HR status of primary LCIS was differentially correlated with types of CBCs. Women with HR- LCIS had a much higher risk of second invasive CBCs compared to those with HR+ LCIS (Fig 2B, *P*<0.001), whereas there was no statistical difference in risk of second ipsilateral carcinoma in situ between the two groups of women (Fig 2C, *P* = 0.855).

Multivariable-adjusted analyses found that women with primary HR+ LCIS had significantly lower risks of both second total CBCs and invasive CBCs compared to those with HR- LCIS (Table 3, total CBCs when stratified by laterality and surgery type: hazard ratio 0.340, 95% CI 0.228–0.509, *P*<0.001; invasive CBCs: hazard ratio 0.172, 95% CI 0.108–0.274, *P*<0.001). In addition, patients treated with surgery showed a lower risk of second total or invasive CBCs compared to those with no surgical therapy (Table 3). Similar to IBCs, women diagnosed with LCIS after 2000 had significantly lower risks of total CBCs and invasive CBCs compared with those who was diagnosed between 1998 and 2000 (Table 3).

**Table 3:** Impact of HR status of first primary LCIS, and other demographic, clinicopathologic or treatment characteristics on risks of second contralateral breast cancers by multivariable-adjusted analyses (n = 10304).

Factors	Total CBC			Invasive CBC		
	Hazard ratio	95% CI	*P*	Hazard ratio	95% CI	*P*
HR						
Negative	Reference			Reference		
Positive	0.340	0.228–0.509	<0.001	0.172	0.108–0.274	<0.001
Year of diagnosis						
1998–2000	Reference			Reference		
2001–2004	0.541	0.409–0.717	<0.001	0.547	0.368–0.812	0.003
2005–2007	0.611	0.433–0.863	0.005	0.573	0.354–0.926	0.023
Histologic grade						
Well	Reference			Reference		
Moderately	0.855	0.641–1.141	0.288	0.810	0.547–1.198	0.291
Poorly/undifferentiated	0.770	0.497–1.192	0.241	0.455	0.228–0.911	0.026
Unknown	0.478	0.335–0.680	<0.001	0.339	0.201–0.570	<0.001
Tumor size						
≤2cm	Reference			Reference		
2-5cm	1.072	0.808–1.424	0.629	1.012	0.669–1.531	0.954
>5cm	1.438	0.867–2.384	0.159	1.443	0.706–2.946	0.314
Unknown	19.562	11.877–32.219	<0.001	24.614	13.245–45.739	<0.001
Surgery for first LCIS						
No surgery	/	/	/	Reference		
BCS	/	/	/	0.181	0.064–0.509	0.001
Mastectomy	/	/	/	0.225	0.080–0.632	0.005

Abbreviations: HR, hormone receptor; LCIS, lobular carcinoma in situ; CBC, contralateral breast cancer; CI, confidence interval; BCS, breast-conserving surgery

## Discussion

This population-based study, the first and largest dataset addressing therisks of second breast cancers among LCIS survivors according to HR status to date, demonstrated that the risk of second invasive breast cancers was significantly increased in women with HR- first primary LCIS compared to those with HR+ LCIS after adjustment for demographic, clinicopathologic, and treatment factors, regardless of whether the outcome was ipsilateral or contralateral breast cancer. And patients with HR- first LCIS had a significantly higher risk of second total CBCs compared to those with HR+ LCIS as well.

These findings expand the limited body of literature on the risk of second breast cancers after primary LCIS because the current study is the first report using SEER data quantified risks of subsequent breast cancers among LCIS survivors according to HR status of primary LCIS. Prior studies only focused on the comparison of second breast tumor risks between patients with primary LCIS and those with DCIS, or between LCIS women with and without chemoprevention[8,6,7,5,19,20]. Similar to the disparity in second breast cancer risk between women with HR+ and HR- first primary LCIS observed in this study, patients with different HR status of primary invasive ductal or lobular breast cancers also been reported to have distinct risks of second contralateral breast cancer [14–16]. Analogously, these studies investigated that HR- invasive breast cancer women had a significantly higher risk of subsequent CBCs than women with HR+ invasive breast cancer. Preclinical studies have found that breast cancer stem cells show an HR- phenotype [21,22], HR- breast cancer patients might be prone to carcinogenesis early in the breast cell maturation process. Another explanation for the higher risk of second breast cancers after HR- LCIS is that some of these women may carry BRCA mutations. 60% to 90% of BRCA1-associtaed breast cancers are HR negative, and these patients have a much higher risk for developing a second breast cancer [23–25].

Interestingly, we observed that risks of second CBCs and IBCs varied by year of first LCIS diagnosis. After adjustment for other demographic,clinicopathologic and treatment factors, women who were diagnosed after the year of 2000 had significantly lower risk of subsequent breast cancers compared with those diagnosed between 1998 and 2000. This reduced second breast cancer risk after primary LCIS diagnosis over time may be resulted in by the increased use of chemoprevention for LCIS patients in 2000s after reports of National Surgical Adjuvant Breast and Bowel Project (NSABP) P-1 and P-2 trials [26,27]. Unfortunately, it was not possible for us to further assess the exact impact of chemoprevention on the risk of second breast cancers due to the lack of chemoprevention information in SEER database. In addition, our observation that the significantly reduced subsequent breast cancer risk after 2000 could explain why the 5-year and 10-year cumulative incidences in this study were slightly lower than those incidences reported in prior study using SEER data (1973–1998) [17]. Because the prior SEER study included women who were diagnosed for primary LCIS between 1973 and 1998, these women may have higher risk of second breast cancers compared to the patients in our study, most of (78.5%) whom were diagnosed after the year of 2000.

In the current study, the risk of second IBCs among LCIS survivors varied by race. Black women had a 2-fold risk of developing subsequent ipsilateral breast tumors than white women. This is consist with prior analyses using SEER database to compare the risks of second breast cancers after first primary DCIS or invasive ductal and lobular breast cancers between blacks and whites [28,29,16]. Therefore, black women with LCIS may need more follow-up and MRI-based breast screening. We have not found any statistical differences in second CBC risk between black and white women in this study, however, further studies are needed to help better understand the impact of race on risk of second CBCs after first primary LCIS.

Although this is the first population-based study defining the risks of second breast cancers among LCIS survivors according to HR status, and it affords large statistical power and representative results, some limitations warrant consideration. Similar to other studies that relied on SEER database, the lack of information on family cancer history, several critical lifestyle and clinicopathological characteristics (diet, hormone replacement, body mass index (BMI), surgical margin status, etc), inherited genetic mutations, and chemoprevention for first LCIS limited our ability to further assess the risk of second breast cancers. Moreover, a general limitation for population-based study is the possibility of underreporting and imperfect ascertainment. The incidences of second breast cancers are generally underestimated. In addition, the pathology and HR reporting particularly in the early part of the study series might not be accurate, which may have impact on some of the results. The lower percentage of HR- cases after 2004 and the reduced second breast cancer risks after primary LCIS diagnosis in later cohorts could be partially explained by improved immunohistochemistry testing and more accurate pathology review over time.

In conclusion, our finding that women with a first primary HR- LCIS have significantly elevated risk of developing second breast cancers has important implications for routine clinical management. HR- LCIS women may also need chemoprevention and more intensive post-treatment follow-up. Furthermore, the observation that black women with LCIS had a 2-fold risk of developing subsequent IBCs than white women suggests that black women with LCIS warrant more surveillance as well. Further studies should focus on identifying the differences of genetic and biological characteristics that contribute to outcome disparities between HR- and HR+ LCIS, in order to target screening, prevention, as well as treatment strategies more effectively.

## Supporting information

S1 Dataset Raw data that underlying the findings of the current study. (DTA)

## Abbreviations

AIC: Akaike information criterion
BMI: body mass index
CBCs: contralateral breast cancers
CI: confidence interval
DCIS: ductal carcinoma in situ
ER: estrogen receptor
HR: hormone receptor
IBCs: ipsilateral breast cancers
IBTR: ipsilateral breast tumors
ICD-O-3: International Classification of Diseases for Oncology, 3rd edition
LCIS: lobular carcinoma in situ
NSABP: National Surgical Adjuvant Breast and Bowel Project
PR: progesterone receptor
SEER: Surveillance, Epidemiology and End Results
